# Supplementary material for: Arctic closure as a trigger for Atlantic overturning at the Eocene-Oligocene Transition
Source: Nat Commun. 2019 Aug 22;10:3797. doi: 10.1038/s41467-019-11828-z (PMC6706372; doi:10.1038/s41467-019-11828-z)
Supplement: Supplementary file 1 — Supplementary Information [file 41467_2019_11828_MOESM1_ESM.pdf]

Supplementary Information

Arctic closure as a trigger for Atlantic overturning at the Eocene-Oligocene Transition

Hutchinson, D. K. et al.

Smelror et al., 2009, *Geologic History of the Barents Sea*

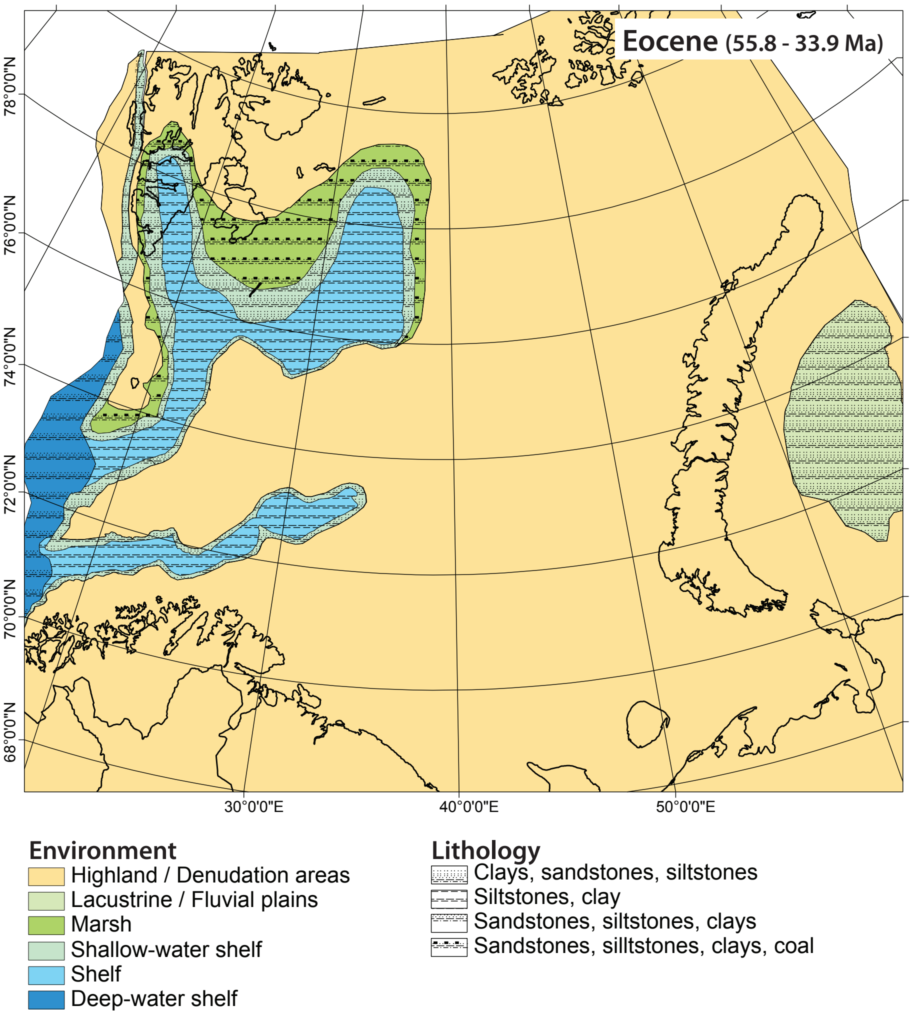

Lasabuda et al., 2018, *Marine and Petroleum Geology*

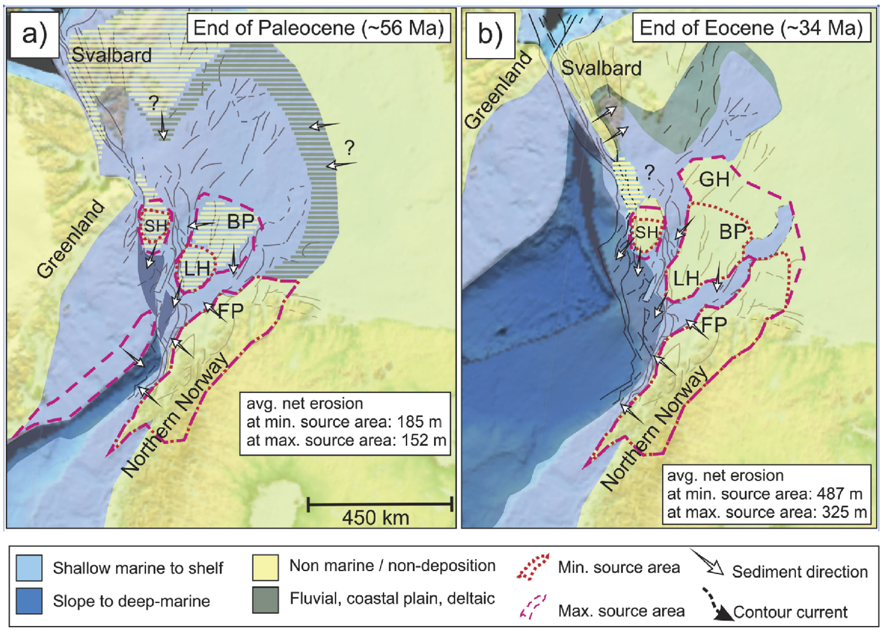

Supplementary Figure 1: (top) Depositional environments of the Barents Sea during the Eocene, where white represents no data, reproduced with permission from Smelror et al[1]. (bottom) Early and late Eocene reconstructions of the Barents Sea, dashed lines represent a transition zone between types, reproduced with permission from Lasabuda et al[2].

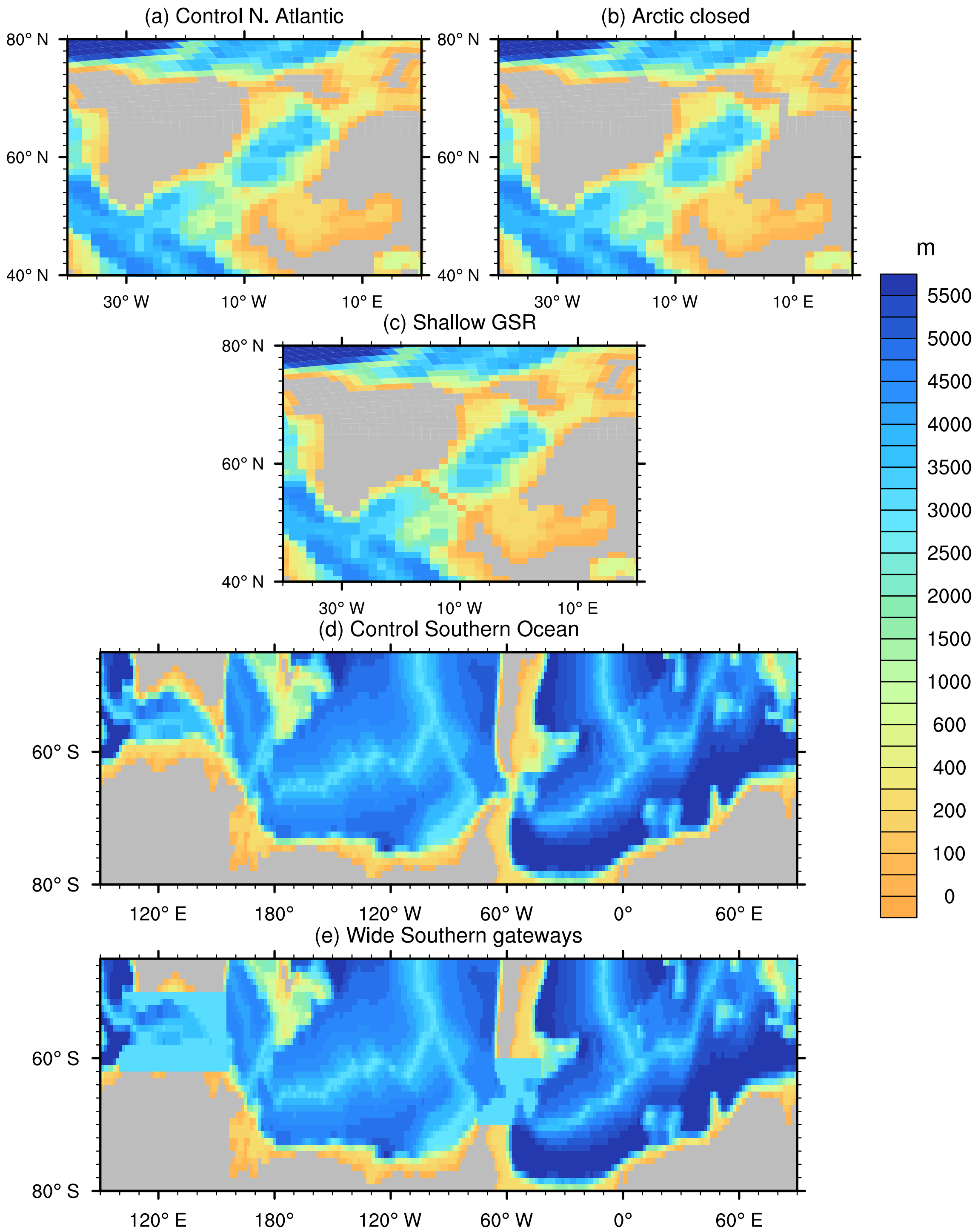

Supplementary Figure 2: North Atlantic bathymetry in (a) the control experiment, (b) Arctic gateway closed, and (c) shallow (25 m) Greenland-Scotland Ridge (GSR); and Southern Ocean bathymetry in the (d) control run and (e) wide Southern gateways experiment.

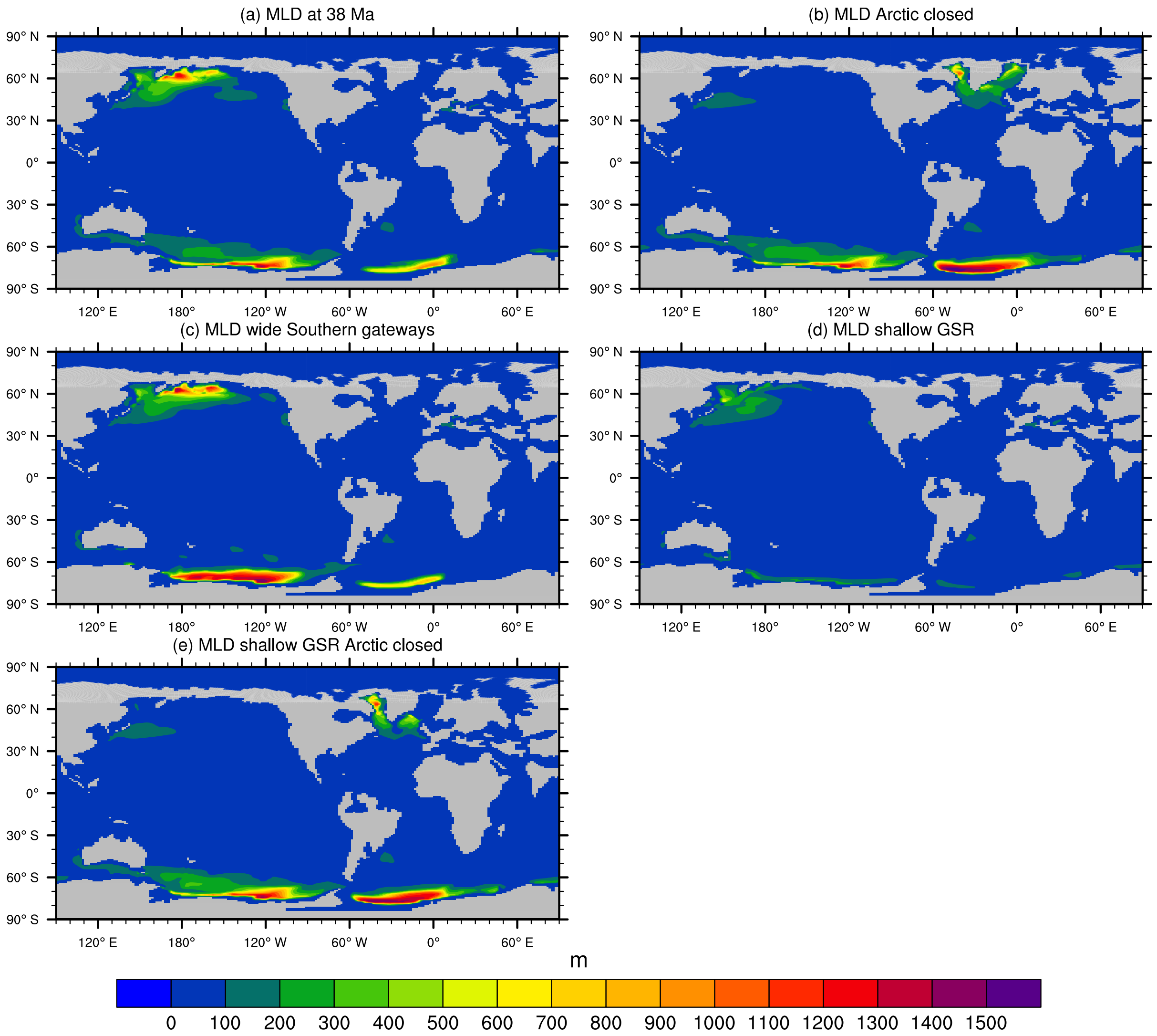

Supplementary Figure 3: Annual Mixed layer depths (MLD) in the following simulations: (a) the control experiment, (b) Arctic-Atlantic gateway closed, (c) wide Southern Ocean gateways, (d) shallow (25 m) Greenland-Scotland Ridge (GSR), and (e) Arctic gateway closed with a shallow GSR.

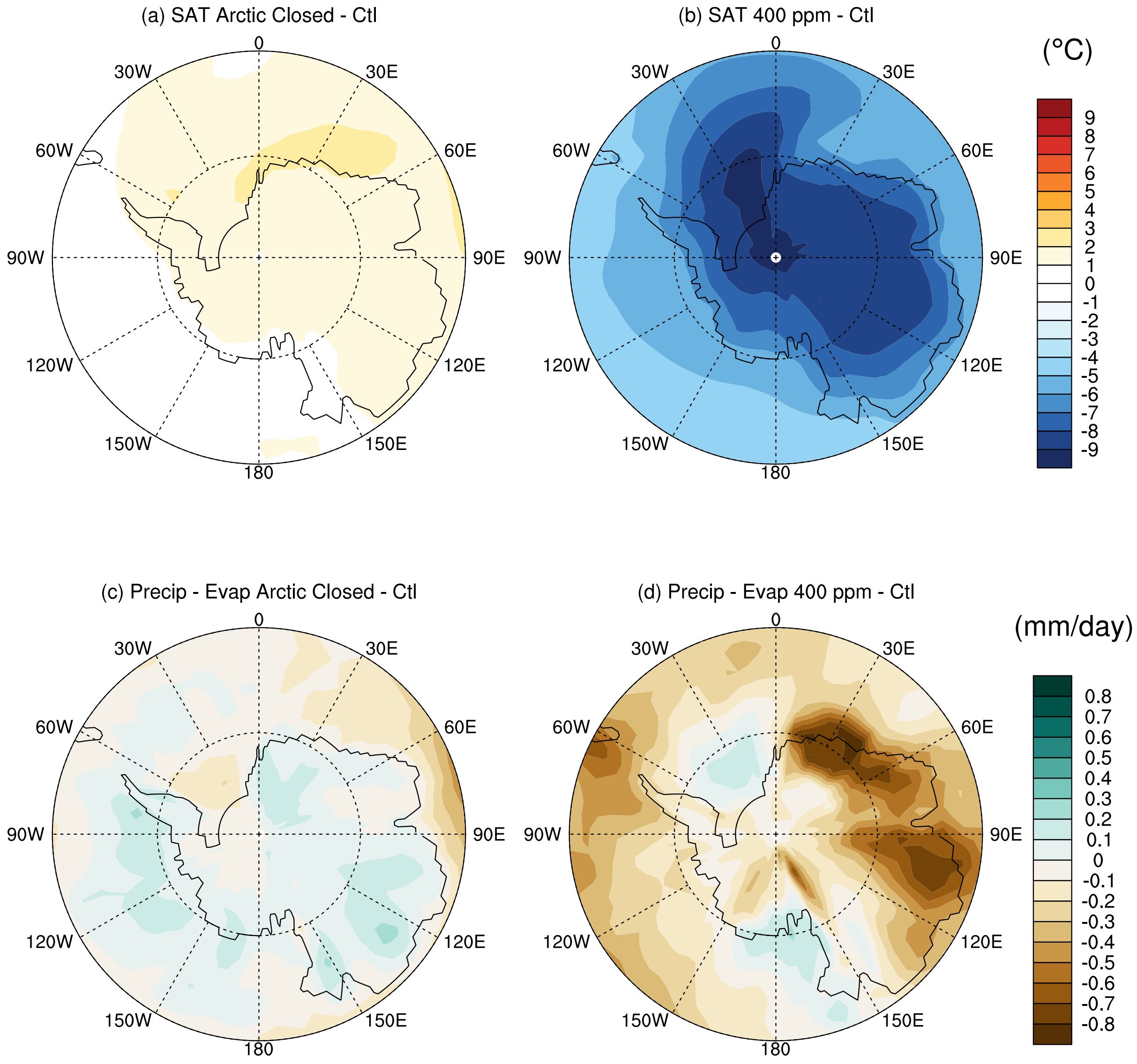

Supplementary Figure 4: Changes over the south pole for surface air temperature (SAT), showing (a) Arctic closed minus control, (b) 400 ppm minus control, and precipitation minus evaporation differences between (c) Arctic closed minus control, and (d) 400 ppm minus control. The 400 ppm simulation[3] has half the CO_2 of the control run (800 ppm).

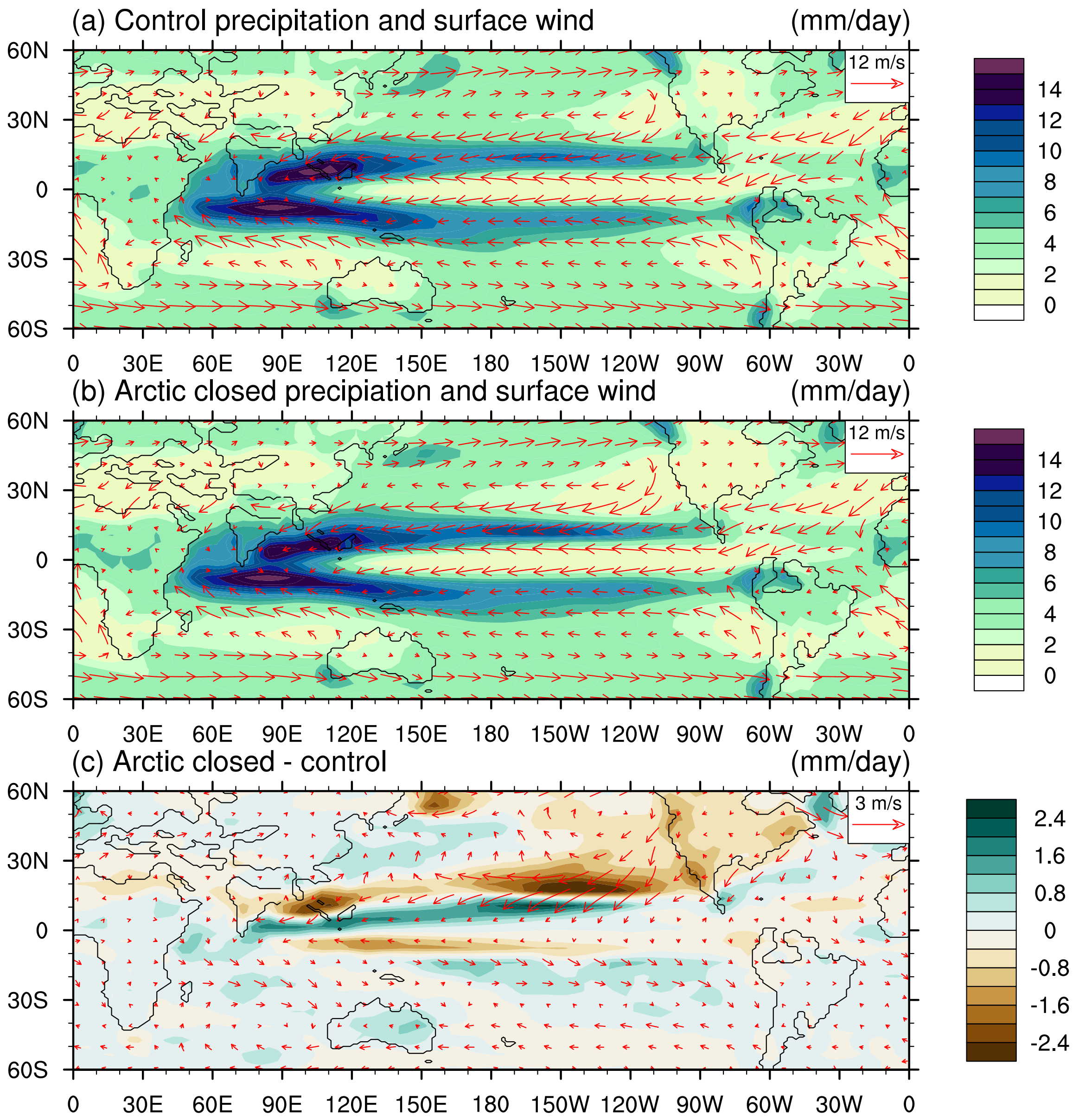

Supplementary Figure 5: Intertropical convergence zone (ITCZ) as depicted by precipitation maxima (contours), with surface wind vectors overlaid. (a) Control experiment, (b) Arctic closed, (c) Arctic closed minus control.

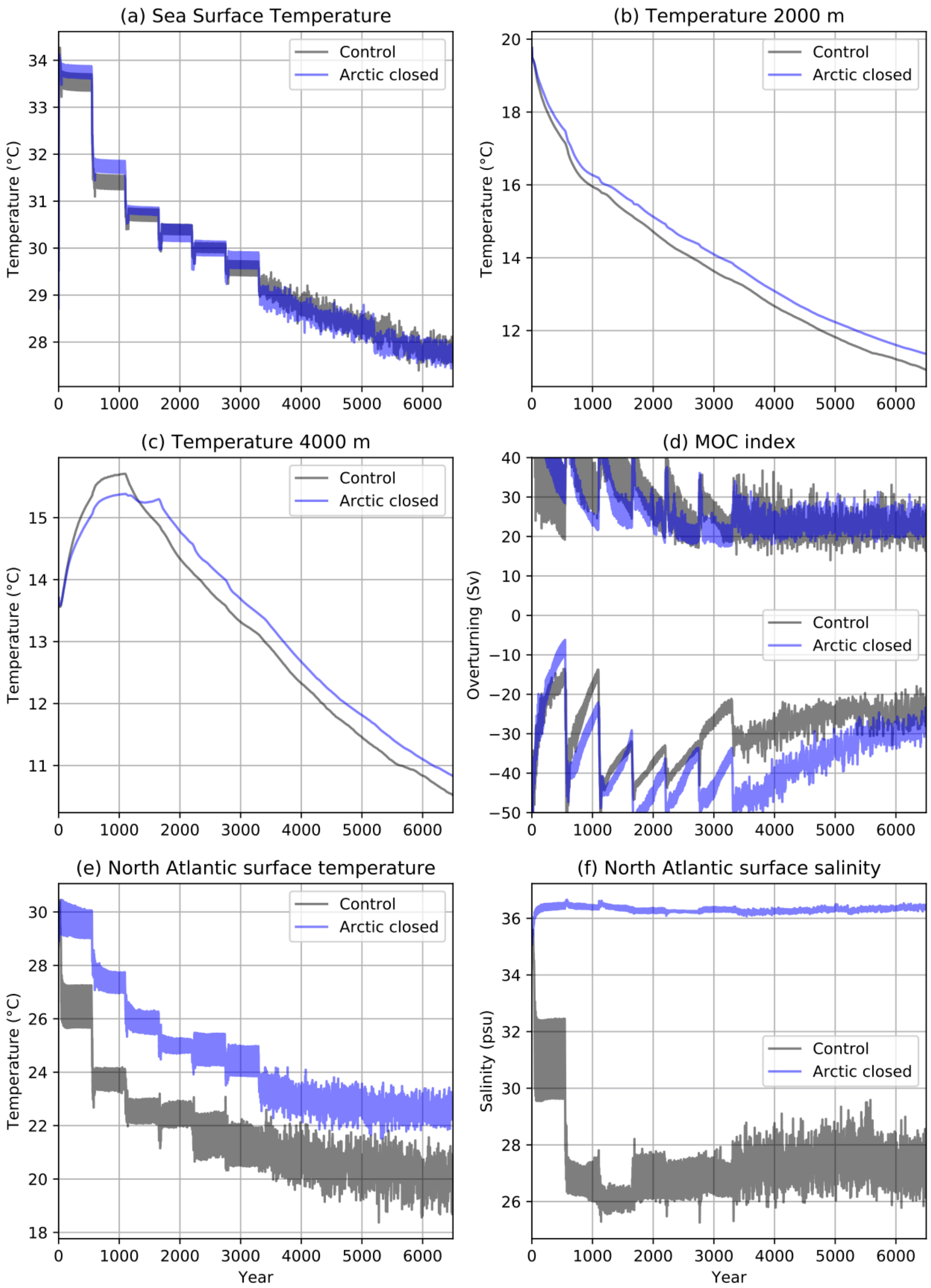

Supplementary Figure 6: Spinup of ocean temperature at (a) the surface (SST), (b) 2000 m depth, (c) 4000 m depth; (d) meridional overturning circulation (MOC) indices, positive indicates northern cell, negative indicates southern cell, (e) North Atlantic sea surface temperature from a sample region between 41 to 51 °N and 37 to 11°W, (f) North Atlantic surface temperature from the same region as (e). This region lies just south of Greenland and approximately spans the width of the Atlantic in these latitudes.

Supplementary References

1. Smelror, M., Petrov, O. V., Larssen, G. B. & Werner, S. C. *Geological history of the Barents Sea*. (Geological Survey of Norway, Trondheim, 2009).
2. Lasabuda, A., Laberg, J. S., Knutsen, S.-M. & Høgseth, G. Early to middle Cenozoic paleoenvironment and erosion estimates of the southwestern Barents Sea: Insights from a regional mass-balance approach. *Mar. Pet. Geol.* **96**, 501–521 (2018).
3. Hutchinson, D. K. *et al.* Climate sensitivity and meridional overturning circulation in the late Eocene using GFDL CM2.1. *Clim. Past* **14**, 789–810 (2018).
